# Public health round-up

**DOI:** 10.2471/BLT.18.010418

**Published:** 2018-04-01

**Renewed “health for all” call to mark WHO’s 70 years**

WHO Director-General Dr Tedros Adhanom Ghebreyesus called on world leaders to live up to the pledges they made, when they agreed on the sustainable development goals in 2015. The theme of World Health Day on 7 April this year, WHO’s 70th anniversary, is universal health coverage. The World Health Organization (WHO) was founded on 7 April 1948. bit.ly/2tH0Jyf

**Figure:**
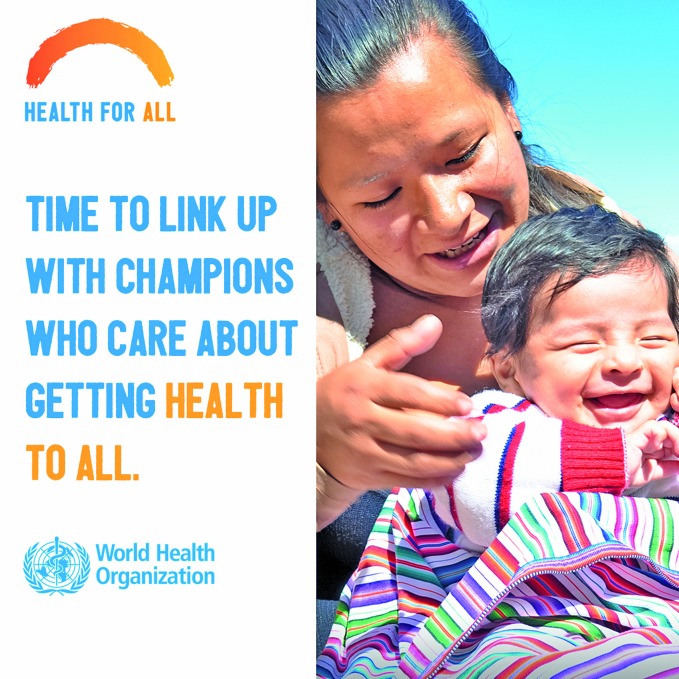
WHO

## Baby friendly initiative 

WHO and the United Nations Children’s Fund (UNICEF) release new guidance this month on how health decision-makers can ensure that mothers in all countries receive the optimal care and support they need to successfully breastfeed their babies. 

The Baby Friendly Hospital Initiative (BFHI) was launched by the two agencies in 1991 to provide guidance to maternity and newborn services on how best to support breastfeeding. 

Almost all countries have implemented the initiative.  However, more than a quarter of a century later, coverage of the initiative at a facility level is low. As of 2017, only 10% of infants were born in a baby-friendly facility.

The initiative is based on the *Ten steps to successful breastfeeding* issued in 1989. The updated guidance reflects new evidence, proposing ways to overcome barriers to BFHI implementation and indicators to measure progress in its implementation. 

*Protecting, promoting and supporting breastfeeding in facilities providing maternity and newborn services: Implementing the revised Baby-friendly Hospital Initiative 2018 *recommends institutional procedures to ensure that care is delivered consistently and ethically, and standards for the individual care of mothers and infants. 

The new guidance calls for the full application of the *International code of marketing of breast-milk substitutes* and related World Health Assembly resolutions, as well as monitoring of adherence to clinical practices on infant feeding policies.

It recommends revisions to the national implementation of the BFHI, including scaling up to universal coverage and ensuring sustainability. It envisages fuller integration of the BFHI into the health-care system, so that all facilities in a country, and not just a select few, implement the Ten Steps. 

Countries are called upon to fulfil nine key responsibilities through a national BFHI programme. These responsibilities include establishing or strengthening a national coordination body, integrating the Ten Steps into national policies and standards, and improving the capacity of all health-care professionals.

The updated guidance is the result of a process WHO and UNICEF began in 2015 to reinvigorate the BFHI programme. 

bit.ly/2DosfA5

## Regulating tobacco products

Tobacco products are one of the few types of consumer products that are widely available but under-regulated in terms of their contents, design features and emissions, according to new WHO guidance. 

*Tobacco product regulation: building laboratory testing capacity*, released last month during the 17th World Conference on Tobacco or Health, in Cape Town, provides a guide for regulators and policymakers on tobacco product testing and how to use data from such tests to support regulation.

The guide covers what is needed to measure nicotine, the primary addictive component of tobacco products, as well as toxic and carcinogenic substances in tobacco product contents and emissions, such as tobacco-specific nitrosamines and polynuclear aromatic hydrocarbons.

“Most countries are reluctant to introduce regulations in this area because of the technical complexity and lack of appropriate guidance,” said Dr Douglas Bettcher, Director of the Department of Prevention of Noncommunicable Diseases. 

“This easy-to-follow guide simplifies the process of testing and how to build laboratory testing capacity, irrespective of a country’s starting point or journey in the implementation of Article 9 and 10 of the WHO Framework Convention on Tobacco Control (WHO FCTC),” he said. 

Article 9 addresses the regulation of the contents and emissions of tobacco products and article 10 addresses the regulation of tobacco product disclosures on the contents and emissions.

The guidance, which updates a 2004 set of recommendations, provides options for building laboratory testing capacity for tobacco products. These options include developing or improving a tobacco testing laboratory, using an existing internal laboratory, contracting an external laboratory and making use of the support mechanisms that are available. 

The WHO Tobacco Laboratory Network is one such support mechanism, others include WHO technical and regulatory groups in the area of tobacco product regulation. 

bit.ly/2FDsEAu

## Yellow fever vaccination

Brazil reported 723 confirmed human cases of yellow fever, including 237 deaths between 1 July 2017 and 28 February 2018 as of 12 March, an increase compared with the 576 confirmed cases, including 184 deaths, reported during the same period a year before. 

Yellow fever is an acute viral haemorrhagic disease transmitted by infected mosquitoes. The increase in the disease is probably due to the virus circulating in densely populated areas, where yellow fever vaccination was not previously recommended.

Since September of last year, when cases of yellow fever in humans, as well as apes were confirmed in São Paulo State, Brazil’s national authorities have stepped up the vaccination response. 

In January, the national authorities launched a vaccination campaign providing fractional and standard doses targeting around 23 million people in three states: Rio de Janeiro, São Paulo and Bahia. By the end of February, about 5.5 million of them (23%) had been vaccinated.

Studies show that one fifth of the regular dose provides full immunity against the disease for at least 12 months. Fractional dosing is the recommended strategy to control an outbreak in highly populated areas to avoid vaccine shortages.

Ensuring that everyone who is at risk of yellow fever is vaccinated is one of the goals of the Global Strategy to Eliminate Yellow Fever Epidemics partnership, led by WHO, UNICEF and Gavi, the Vaccine Alliance. 

Demand for the yellow fever vaccine has increased following a resurgence of yellow fever epidemics in Africa and Latin America driven by changing epidemiology, population movement, climate change and other factors. 

Last month the International Coordination Group (ICG) on Vaccine Provision for Yellow Fever reported that in 2016, 13 of 15 requests for the provision of yellow fever vaccine for outbreaks were approved. In addition 30.2 million doses, more than one third of global output, were released for use in Angola, the Democratic Republic of Congo (DRC) and Uganda that year. 

The ICG said that 6.8 million doses were dispatched to Brazil and Nigeria in 2017, and that the 2016 and 2017 epidemic seasons saw major yellow fever outbreaks in Angola, Brazil, DRC and Nigeria. The 2016 outbreaks in Africa depleted the emergency stockpile on two occasions, and vaccines earmarked for routine preventive use were diverted for emergency response. 

bit.ly/2GkCh8o

**Cover photo**

A Nigerian refugee in Chad unloads his morning's catch.

**Figure:**
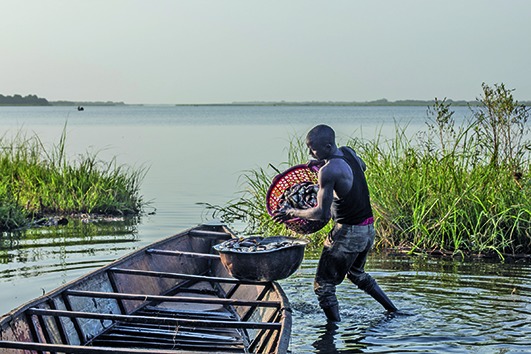
© UNHCR/Oualid Khelifi

## New HIV guidelines 

WHO released new guidelines on the diagnosis, prevention and management of cryptococcal disease (including meningitis), a serious opportunistic infection and major cause of morbidity and mortality in people with HIV infection with advanced disease.

Cryptococcal meningitis accounts for an estimated 15% (181 000) of all HIV-related deaths globally. Mortality from the disease is highest in low-income countries.

Delays in diagnosis, as a result of limited access to rapid diagnostic assays and lumbar puncture (a medical testing procedure), and the limited availability and the high cost of first-line antifungal drugs are major contributors to this high mortality.

*Guidelines for the diagnosis, prevention and management of cryptococcal disease in HIV-infected adults, adolescents and children* provides new and updated recommendations in several areas. 

These recommendations cover the optimal approach to diagnosing cryptococcal meningitis, strategies for preventing invasive cryptococcal disease through cryptococcal antigen screening and pre-emptive therapy with the anti-parasitic medication, fluconazole.

bit.ly/2Dn59dd

## Kenya certified dracunculiasis free

WHO certified Kenya free of dracunculiasis transmission (guinea-worm disease) last month following a recommendation by the International Commission for the Certification of Dracunculiasis Eradication.

Kenya is one of 20 countries where the disease was endemic in the 1980s, when the eradication goal was set. Today it is endemic in three: Chad, Ethiopia and Mali. 

To be declared dracunculiasis free, a country must show that it has stopped transmission of the disease by reporting zero indigenous cases and guinea-worm infected animals through active surveillance for at least three consecutive calendar years. 

It must also show that the risk of re-introduction of the disease is minimal through a robust surveillance system that can detect and contain any imported case, should it occur.

Seven countries, including the three endemic countries, remain to be certified before WHO can declare that the world is dracunculiasis free. 

In 2017, dracunculiasis was reported in Chad and Ethiopia, each with 15 cases. This brings the cumulative total in 2017 to 30 human cases – five cases more than the 2016 total. 

Mali has reported zero human cases since November 2015.

South Sudan and Sudan are in the pre-certification stage. South Sudan reported zero human cases of the disease during the whole of 2017 and the last three indigenous cases in Sudan were reported in 2013. 

Sudan is currently strengthening its surveillance system to further minimize the risk of disease reintroduction and meet the certification criteria. 

WHO is supporting countries in their dracunculiasis elimination efforts with the help of the Carter Center, UNICEF, the WHO Collaborating Centre at the United States Centers for Disease Control and Prevention and other partners. 

bit.ly/2paLRTh

**Looking ahead**

25 April – World Malaria Day

24-30 April – World Immunization Week

21–26 May – Seventy-first World Health Assembly

31 May – World No Tobacco Day. Theme: Protected Together #VaccinesWork

